# Wurtzite-derived ternary I–III–O_2_ semiconductors

**DOI:** 10.1088/1468-6996/16/2/024902

**Published:** 2015-03-17

**Authors:** Takahisa Omata, Hiraku Nagatani, Issei Suzuki, Masao Kita

**Affiliations:** 1 Division of Materials and Manufacturing Science, Graduate School of Engineering, Osaka University, 2-1 Yamadaoka, Suita, Osaka 565-0871, Japan; 2 Department of Mechanical Engineering, Toyama National College of Technology, 13 Hongo-machi, Toyama 939-8630, Japan

**Keywords:** II–VI semiconductor, I–III–VI_2_ semiconductor, oxide semiconductor, 72.80.Ey, 78.40.Fy, 81.05.Dz

## Abstract

Ternary zincblende-derived I–III–VI_2_ chalcogenide and II–IV–V_2_ pnictide semiconductors have been widely studied and some have been put to practical use. In contrast to the extensive research on these semiconductors, previous studies into ternary I–III–O_2_ oxide semiconductors with a wurtzite-derived *β*-NaFeO_2_ structure are limited. Wurtzite-derived *β*-LiGaO_2_ and *β*-AgGaO_2_ form alloys with ZnO and the band gap of ZnO can be controlled to include the visible and ultraviolet regions. *β*-CuGaO_2_, which has a direct band gap of 1.47 eV, has been proposed for use as a light absorber in thin film solar cells. These ternary oxides may thus allow new applications for oxide semiconductors. However, information about wurtzite-derived ternary I–III–O_2_ semiconductors is still limited. In this paper we review previous studies on *β*-LiGaO_2_, *β*-AgGaO_2_ and *β*-CuGaO_2_ to determine guiding principles for the development of wurtzite-derived I–III–O_2_ semiconductors.

## 1. Introduction

Oxide semiconductors have advanced much over the last two decades and currently play an important role in inorganic functional materials. They are used in optoelectronic devices; for example, indium tin oxide, tin oxide and zinc oxide are used as transparent electrodes [1–3] and amorphous indium–gallium–zinc oxide is used in transparent thin-film transistors [4]. Their transparency for visible light is a relatively passive optoelectronic function that comes from their wide band gap and this is used in their applications. Among the oxide semiconductors, ZnO is a II–VI semiconductor with a wurtzite structure [5, 6] and it is an attractive material for use in active optoelectronic devices such as light emitting diodes (LEDs), lasers and photovoltaics because of the nature of its direct band gap [7]. However, the binary oxide semiconductors that possess the wurtzite structure are limited to ZnO and the carcinogenic BeO [8]. Therefore, the band gap engineering of ZnO over a wide energy range is difficult compared to II–VI chalcogenide and III–V pnictide semiconductors. The band gap (*E*_*g*_) of ZnO is usually controlled by alloying with rock-salt-type MgO [9–11]. The adjustable energy range of these alloys is limited to the range from 3.4 to 4.0 eV because the alloying range is small and *x* < 0.4 in *x*MgO–(1 − *x*)ZnO due to structural differences between MgO and ZnO [9, 10]. Consequently, the usable wavelength region of devices using ZnO is limited to the near-ultraviolet region.

**Figure 1.**
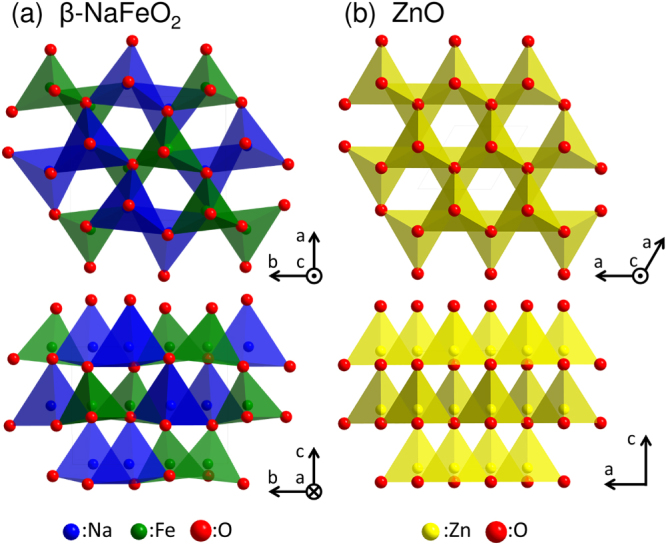
Schematic of (a) *β*-NaFeO_2_ and (b) wurtzite structures.

For chalcogenide semiconductors, ternary I–III–VI_2_ chalcopyrite semiconductors such as CuInS_2_ and CuGaSe_2_ have been extensively studied [12–14]. The band gap engineering of II–VI zincblende chalcogenides alloyed with I–III–VI_2_ chalcopyrite chalcogenides has also been studied [15–21]. The chalcopyrite structure is a binary zincblende superstructure [22] where two divalent cations in a II–VI zincblende material are replaced by a monovalent and a trivalent cations. A similar superstructure with a specific relationship between the zincblende and chalcopyrite structures is found in the wurtzite structure of wurtzite-derived *β*-NaFeO_2_ structure [23]. Here, monovalent and trivalent cations occupy the divalent cation site in the wurtzite structure, and both cations have a four-fold tetrahedral coordination to oxygen atoms in addition to an ordered arrangement, as schematically shown in figure 1. Based on the structural similarity between *β*-NaFeO_2_ and the wurtzite structure, the alloying of ZnO with ternary oxides possessing the *β*-NaFeO_2_ structure is expected to occur over a wide composition range. This is preferred over alloying with binary oxides that possess a rock-salt structure such as MgO and CdO. The possible energy range of the band gap engineering of ZnO is expected to increase upon alloying with the ternary oxides. Whereas research into the chalcogenide I–III–VI_2_ chalcopyrite semiconductors has been much active, and even ternary II–IV–N_2_ nitrides such as ZnGeN_2_ and ZnSnN_2_ have recently been studied extensively [24–26], ternary I–III–O_2_ semiconductors with a wurtzite-derived *β*-NaFeO_2_ structure have received little attention.

We have recently focused on ternary I–III–O_2_ semiconductors with a wurtzite-derived *β*-NaFeO_2_ structure and their alloys with ZnO in these circumstances. In particular, the band gap engineering of ZnO in the UV and visible regions has been demonstrated by alloying with *β*-LiGaO_2_ (*E*_*g*_ = 5.6 eV) [27] and *β*-AgGaO_2_ (*E*_*g*_ = 2.2 eV) [28], respectively. We found that *β*-CuGaO_2_, which is a *p*-type semiconductor with a direct band gap of 1.47 eV, is a new ternary wurtzite oxide semiconductor material [29]. In this paper, we briefly review recent advances in ternary I–III–O_2_ semiconductors with a *β*-NaFeO_2_ structure with a focus on our previous studies.

## 2. *β*-LiGaO_2_ and its alloys with ZnO

**Figure 2.**
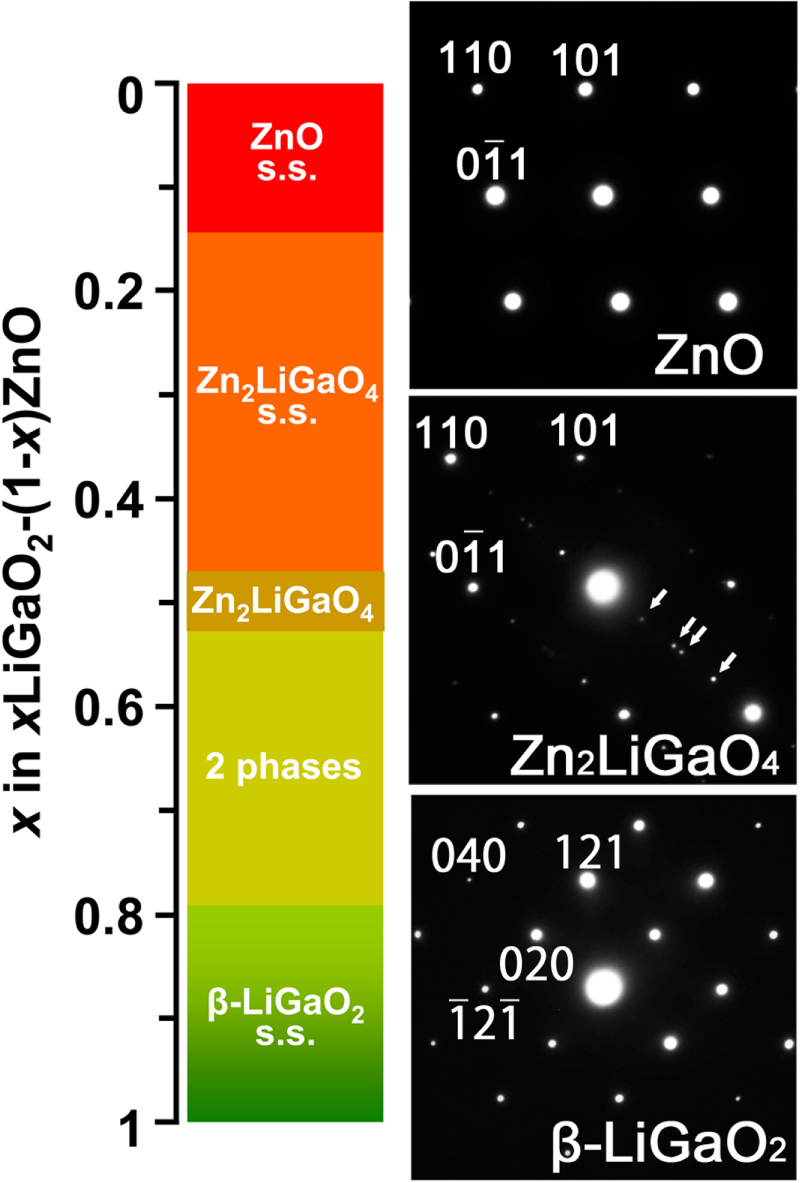
Phase variation in the (1 − *x*)ZnO–*x*(LiGaO_2_)_1/2_ pseudo-binary system together with the selected area electron diffraction (SAED) of ZnO, Zn_2_LiGaO_4_ and *β*-LiGaO_2_. The SAED patterns were recorded for the 〈
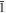
11〉 zone axis of the hexagonal wurtzite structure for ZnO and Zn_2_LiGaO_4_ and the 〈
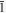
01〉 zone axis of orthorhombic *β*-LiGaO_2_. The arrows in the SAED of Zn_2_LiGaO_4_ indicate superlattice diffractions.

*β*-LiGaO_2_, which possesses a band gap of 5.6 eV, is the best-known wurtzite-derived ternary oxide semiconductor. High purity single crystals several inches long can be grown by the Czochralski method and they can be cleaved to form faces that are lattice-matched to ZnO and GaN [30–32]. It has been studied as a substrate material for ZnO and GaN and as an insulating layer for epitaxially grown ZnO-based multilayers [33]. *β*-LiGaO_2_ has also been studied as a material for use in nonlinear optics [34–36]. We reported on the band gap engineering of ZnO, which included the UV region, upon alloying with *β*-LiGaO_2_ [37, 38]. Figure 2 shows the phase variation in the pseudo-binary alloy system of *x*(LiGaO_2_)_1/2_–(1 − *x*)ZnO. This was determined by the solid state reaction between ZnO and *β*-LiGaO_2_ at 1100 °C. *β*-LiGaO_2_ dissolves in ZnO to form wurtzite-related alloys up to *x* = 0.5. This alloy formation range is much wider than that of the MgO–ZnO system, where the equilibrium solubility limit of MgO in ZnO at 550°–1200 °C is *x* ∼ 0.15 in *x*MgO–(1 − *x*)ZnO [10, 11]. Detailed characterization of the phases reveled that a quaternary wurtzite-derived Zn_2_LiGaO_4_ phase is present at *x* = 0.5 [39]. The Zn_2_LiGaO_4_ is clearly distinguished from the wurtzite phase (the space group of P6_3_mc) and the *β*-LiGaO_2_ phase (the space group of Pna2_1_) because clear superlattice diffractions appear in its powder x-ray diffraction (XRD) and selected area electron diffraction (SAED; figure 2). Its Raman spectrum was also completely different from that of wurtzite and *β*-LiGaO_2_ phases. The cations of Zn^2+^, Li^+^ and Ga^3+^ in Zn_2_LiGaO_4_ may have an ordered arrangement similar to the Li_2_BeSiO_4_ crystal (the space group of P1n1) [40]; however, the crystal structure has not been determined yet because of its incommensurate nature. Powder XRD, SAED and Raman spectra also elucidated that the phase that appeared in the *x*(LiGaO_2_)_1/2_–(1 − *x*)ZnO pseudo-binary system varied upon increasing the alloying level as the wurtzite-type phase for 0≤*x* < 0.2, the Zn_2_LiGaO_4_-type phase for 0.2 ≤ *x* ≤ 0.5 and the *β*-LiGaO_2_-type phase for 0.8 ≤ *x* ≤ 1. The intermediate composition of 0.5 < *x* < 0.8 is a mixture of the Zn_2_LiGaO_4_ and *β*-LiGaO_2_ phases.

**Figure 3.**
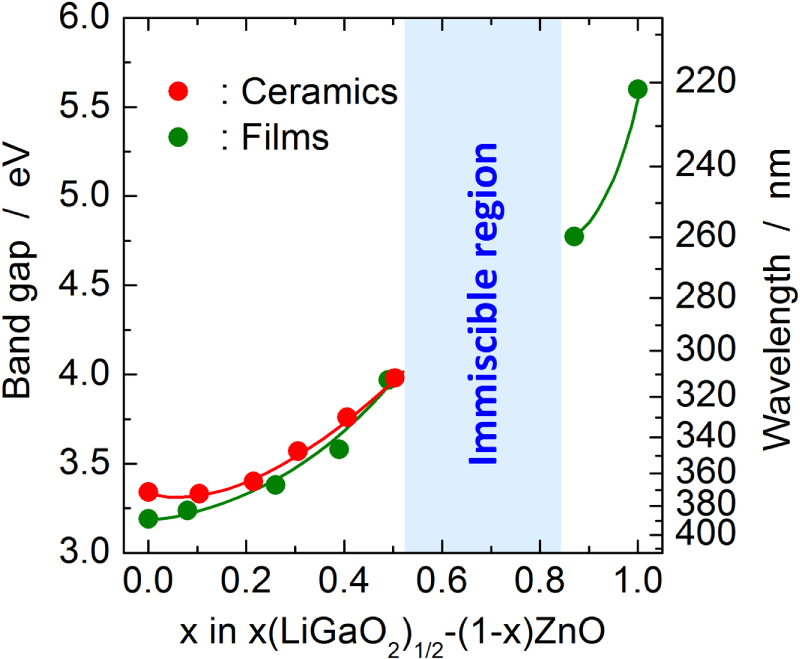
Band gap variation as a function of *x* in the (1 − *x*)ZnO–*x*(LiGaO_2_)_1/2_ pseudo-binary system. The red dots and green dots indicate the band gaps determined for the ceramics and the films, respectively. The blue rectangle indicates a two phase region of Zn_2_LiGaO_4_ and *β*-LiGaO_2_ solid solutions. Although the boundary of the wurtzite-type phase and Zn_2_LiGaO_4_-type phase is between *x* = 0.1 and 0.2, all data are connected by one line, because both the phases are based on the wurtzite structure.

The band gap of ZnO increases to 4.04 eV upon alloying with *β*-LiGaO_2_ as shown in Figure 3 [37, 38]. The largest band gap of the *β*-LiGaO_2_–ZnO alloy system is comparable with that of MgO–ZnO alloy films (∼4.0 eV) that are fabricated under non-equilibrium conditions [9, 10]. The band gap is significantly larger than that of MgO–ZnO alloys fabricated under equilibrium conditions (∼3.5 eV) [10, 11]. *β*-LiGaO_2_ enables a widening of the band gap of ZnO to ∼4 eV under equilibrium conditions because of its high solubility in ZnO, which arises from their structural similarity. Although the change in band gap depending on the alloying level exhibits bowing as observed in figure 3, the extent of the bowing is very small [38]. This also comes from the small lattice mismatch and the chemical mismatch, i.e., the band offset between *β*-LiGaO_2_ and ZnO.

Some theoretical studies about the electronic structure of *β*-LiGaO_2_ have been reported [41–44]. They indicate that *β*-LiGaO_2_ is a direct band gap semiconductor and its valence band (VB) and conduction-band consist mainly of O 2p and Ga 4s states, respectively. This is similar to other oxide semiconductors containing cations with a (*n* − 1)d^10^*n*s^0^ electronic configuration. Recently, first principle calculations of the electronic structure of *x*(LiGaO_2_)_1/2_–(1 − *x*)ZnO alloys were reported by Li and Kuo [45]. They found that the band gaps for all the alloy compositions are direct and a slight band gap bowing between *x* = 0 and 0.5 was evident, which is consistent with experimental observations. The direct band gap nature of these alloys allows their use in optoelectronic applications such as LEDs.

The *n*-type electrical conduction of ZnO is retained in the *x*(LiGaO_2_)_1/2_–(1 − *x*)ZnO alloys up to at least *x* ≤ 0.5. The alloy ceramics sintered under atmospheric conditions are white and they are electrically insulating. When the ceramics are annealed in a hydrogen atmosphere, the color changes to grayish blue because of the plasma oscillation of the conduction electrons, and significant electrical conductivity results. For the 0.38(LiGaO_2_)_1/2_–0.62ZnO alloy ceramic, the electrical conductivity after annealing at 600 °C for 5 h under hydrogen atmosphere was 8.2 Scm^−1^ at room temperature [37]. The fabrication of alloyed thin films of *x*(LiGaO_2_)_1/2_–(1 − *x*)ZnO have also been studied by rf-magnetron sputtering using mixed powders of ZnO and *β*-LiGaO_2_ in an appropriate molar ratio as sputtering target materials [46]. The alloying range and changes in the band gap of the alloy films were almost identical to those observed in alloy ceramics as shown in figure 3. In the optical transmission spectrum of the as-deposited 0.49(LiGaO_2_)_1/2_–0.51ZnO film, a broad absorption in the near-infrared region that is attributable to the plasma oscillation of the conduction electrons was clearly observed. This indicates that the film has significant electrical conduction despite its wide band gap of ∼4.0 eV.

## 3. *β*-AgGaO_2_ and its alloys with ZnO

At the AgGaO_2_ composition, the *α*-AgGaO_2_ with a delafossite structure (the space group P6_3_mc) [47], where the monovalent silver ions are two-fold linearly coordinated to oxygen ions, and the trivalent gallium ions are six-fold octahedrally coordinated to oxygen ions, is a well-known *p*-type and transparent oxide conductor [48]. *β*-AgGaO_2_ with a wurtzite-derived *β*-NaFeO_2_ structure (the space group Pna2a) is probably a metastable phase that can be synthesized by ion-exchange with the precursor *β*-NaGaO_2_ [28, 49]. Previous studies on *β*-AgGaO_2_ have focused on the visible light-driven photocatalytic activity of this material as well as the isostructural *β*-AgAlO_2_ [28, 49–51], because the band gaps of *β*-AgGaO_2_ and *β*-AgAlO_2_ are 2.2 and 2.8 eV, respectively, and the gap is adjustable in the visible region by the alloying of these two materials, i.e., the *β*-Ag(Ga,Al)O_2_ alloy system [52]. The band gap of ZnO has been previously reduced by alloying with toxic CdO; therefore, ZnO cannot be used practically for visible region applications. The band gap of 2.2 eV in less-toxic *β*-AgGaO_2_ [28, 53] is very attractive in terms of the band gap engineering of ZnO into the visible region.

**Figure 4.**
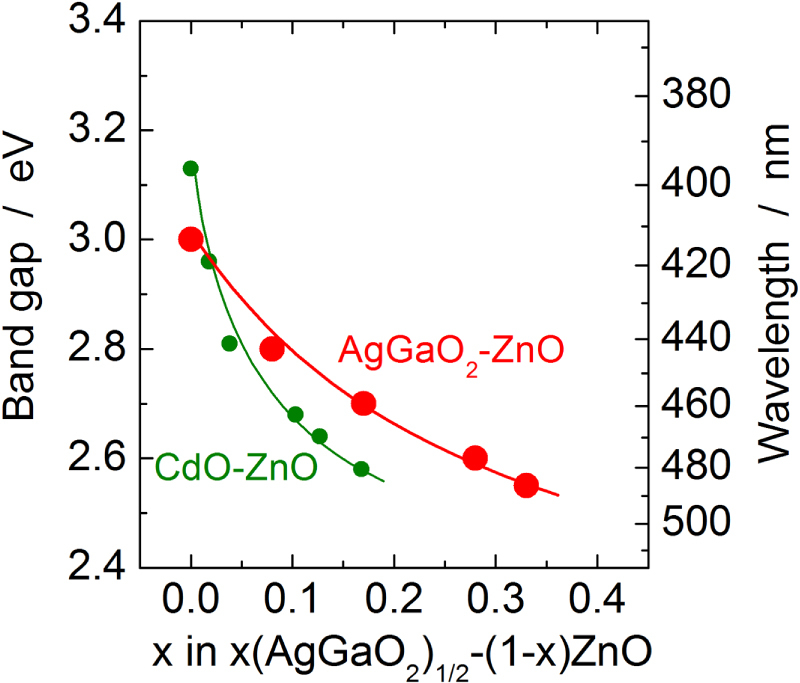
Variation of optical band gap of the (1 − *x*)ZnO–*x*(AgGaO_2_)_1/2_ alloys (red dots and line) as a function of the alloying level, *x*, together with that reported for the (1 − *x*)–*x*CdO alloys (green dots and line) for comparison.

Unlike the *x*(LiGaO_2_)_1/2_–(1 − *x*)ZnO alloys, the *x*(AgGaO_2_)_1/2_–(1 − *x*)ZnO alloys cannot be fabricated by a conventional solid state reaction between ZnO and *β*-AgGaO_2_, because Ag^+^ ions are easily reduced to metallic silver at high temperature. The alloy films are fabricated by rf-magnetron sputtering using mixed ZnO and *β*-AgGaO_2_ powders as target materials [54]. Wurtzite-type alloy films form when *x* ≤ 0.33. This alloying range is wider than that of the *x*CdO–(1 − *x*)ZnO system with *x* < 0.17. This is expected from the structural similarity between ZnO and *β*-AgGaO_2_. Nevertheless, the alloying range is slightly smaller than that of the *β*-LiGaO_2_–ZnO system because of lattice mismatch between ZnO and *β*-AgGaO_2_ (4.6% in the *ab*-plane and 5.2% along the *c*-axis of the wurtzite structure) [54], which is larger than that between ZnO and *β*-LiGaO_2_ (3.0% in the *ab*-plane and 3.8% along the *c*-axis) [37]. Figure 4 shows the change in optical band gap of the *x*(AgGaO_2_)_1/2_–(1 − *x*)ZnO alloys as a function of the alloying level, *x*, together with that reported for the *x*CdO–(1 − *x*)ZnO alloys for comparison [55]. The band gap decreases with an increase in alloying level and was 2.55 eV for *x* = 0.33. Compared with the CdO–ZnO system, the narrowest band gap was approximately the same. In figure 4, comparatively large reduction in the band gap is evident for the composition with a small AgGaO_2_ concentration at *x* ≤ 0.1. However, a near linear decrease occurs with an increase in alloying level. Therefore, band gap bowing in the *β*-AgGaO_2_–ZnO alloys is much smaller than that in the CdO–ZnO system. The large reduction in the band gap during the early alloying stage can be explained by the introduction of the Ag 4d contribution around the valence band maximum (VBM), which mainly consists of the O 2p states in pure ZnO. The introduction of the Ag 4d contribution highly modulates the electronic configuration of the VB of ZnO because of the higher energy of the Ag 4d atomic orbitals compared with that of the O 2p atomic orbitals.

The electronic band structures of *β*-AgGaO_2_ and *β*-AgAlO_2_ were calculated using density functional theory and reported by Maruyama *et al* [49] and Ouyang *et al* [28, 51, 52]. Both materials are indirect band gap semiconductors whereas *β*-LiGaO_2_ is a direct band gap semiconductor. The Ag 4d states significantly contribute to the VBM similarly to the delafossite *α*-AgGaO_2_ and this is expected because of the energy of the Ag 4d atomic orbitals. However, the dispersion of the VB of *β*-AgGaO_2_ is smaller than that of the delafossite *α*-AgGaO_2_. The lower photocatalytic activity of *β*-AgGaO_2_ compared with *α*-AgGaO_2_ can be explained by this small dispersion of the VB, which indicates the heavy effective mass of the holes [49]. Although *β*-AgGaO_2_ is an indirect band gap semiconductor, *x*(AgGaO_2_)_1/2_–(1 − *x*)ZnO alloys where *x* ≤ 0.33 are expected to be direct band gap semiconductors because no discontinuity is observed upon a change in band gap as shown in figure 4. The contribution of the Ag 4d states around the VBM may enable an easier realization of *p*-type electronic conduction for the *β*-AgGaO_2_–ZnO alloys based on the *p*-type conduction of delafossite *α*-AgGaO_2_ [48]. The electrical conduction of *β*-AgGaO_2_ and its alloys with ZnO has not been reported. Future progress in this field is anticipated.

## 4. *β*-CuGaO_2_; a direct and narrow band gap oxide semiconductor

**Figure 5.**
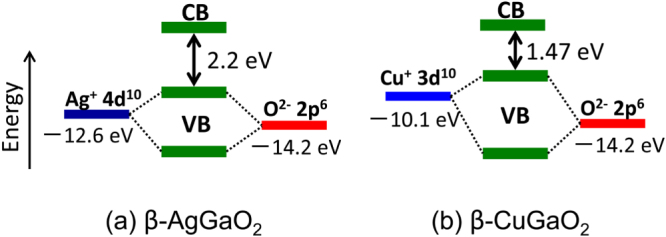
Schematic illustration of the chemical bond between an oxide ion and a monovalent silver or copper ion in (a) *β*-AgGaO_2_ and (b) *β*-CuGaO_2_.

For I–III–O_2_ semiconductors with a delafossite structure, materials containing monovalent copper such as *α*-CuAlO_2_, *α*-CuGaO_2_, and *α*-CuInO_2_ exist in addition to materials containing monovalent silver [56, 57]. However, wurtzite-derived I–III–O_2_ materials with a *β*-NaFeO_2_ structure that contain monovalent copper had not been reported before we began to study the synthesis of *β*-CuGaO_2_. Figure 5 shows a schematic illustration of formation of the chemical bond between an oxide ion and a monovalent silver or copper ion with an (*n* − 1)d^10^*n*s^0^ electronic configuration. The top of the VB is formed by the Ag 4d or Cu 3d states and the O 2p states. Both the O 2p and the Ag 4d or Cu 3d orbitals are fully occupied by electron pairs assuming that the O^2−^, Ag^+^ or Cu^+^ ions and the resulting antibonding level forms the highest occupied level, i.e., the Ag 4d or Cu 3d significantly contributes to the VBM of the materials [58]. Taking the higher energy of the Cu 3d atomic orbitals rather than the Ag 4d atomic orbitals into account [59], the VBM for the wurtzite-derived I–III–O_2_ materials containing the monovalent copper should be higher than that of the materials that contain monovalent silver. This leads to a narrower band gap for the material that contains copper rather than silver when the trivalent cation species are the same. In fact, a comparison between the delafossite *α*-AgGaO_2_ and *α*-CuGaO_2_ semiconductors indicates that the band gap of *α*-CuGaO_2_ is smaller than that of *α*-AgGaO_2_ [48, 60, 61]. Consequently, wurtzite-derived *β*-CuGaO_2_ is expected to have a band gap narrower than that of *β*-AgGaO_2_ and the energy range covered by the wurtzite and its derived oxide semiconductors will expand into the near-infrared region.

**Figure 6.**
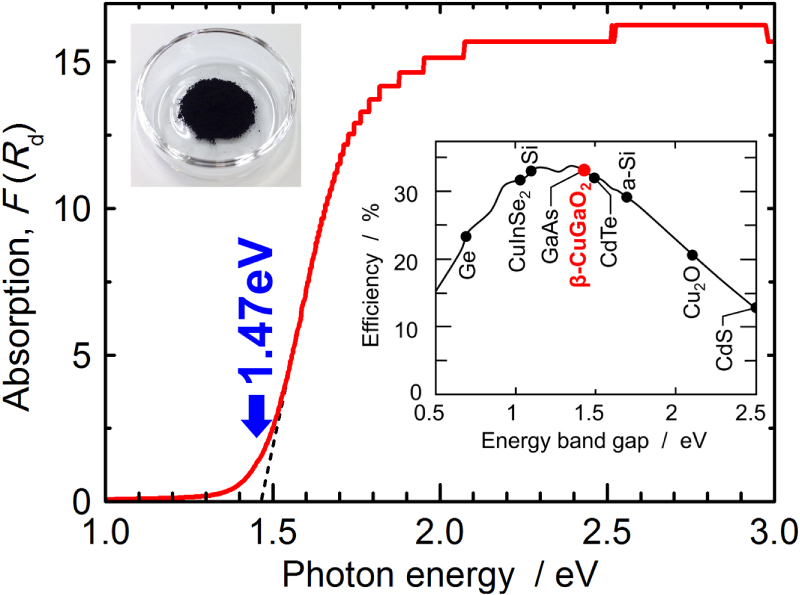
Optical absorption spectrum, *F*(*R*_d_) of *β*-CuGaO_2_ obtained from the diffuse reflection, *R*_d_, using the Kubelka–Munk function. Insets are a picture of powdered *β*-CuGaO_2_ and the theoretical conversion efficiency of a single-junction solar cell as a function of the band gap energy based on the Shockley–Queisser limit using the AM1.5G solar spectrum as the illumination source.

The solid state reaction at high temperatures between Cu_2_O and Ga_2_O_3_ results in delafossite *α*-CuGaO_2_. Wurtzite-derived *β*-CuGaO_2_ (the space group Pna2_1_) can be obtained by an ion-exchange of Na^+^ ions in the precursor *β*-NaGaO_2_ with Cu^+^ ions in CuCl under an evacuated atmosphere to avoid the oxidation of Cu^+^ to Cu^2+^ [29, 62]. *β*-CuGaO_2_ is a black material and its absorption edge appears at 1.47 eV in the near-infrared region as shown in figure 6. Oxide semiconductors are mainly wide band gap materials, and this is an important feature for their use in oxide semiconductors. *β*-CuGaO_2_ is a rare oxide semiconductor with a narrow band gap in the near-infrared region unlike the common oxide semiconductors.

**Figure 7.**
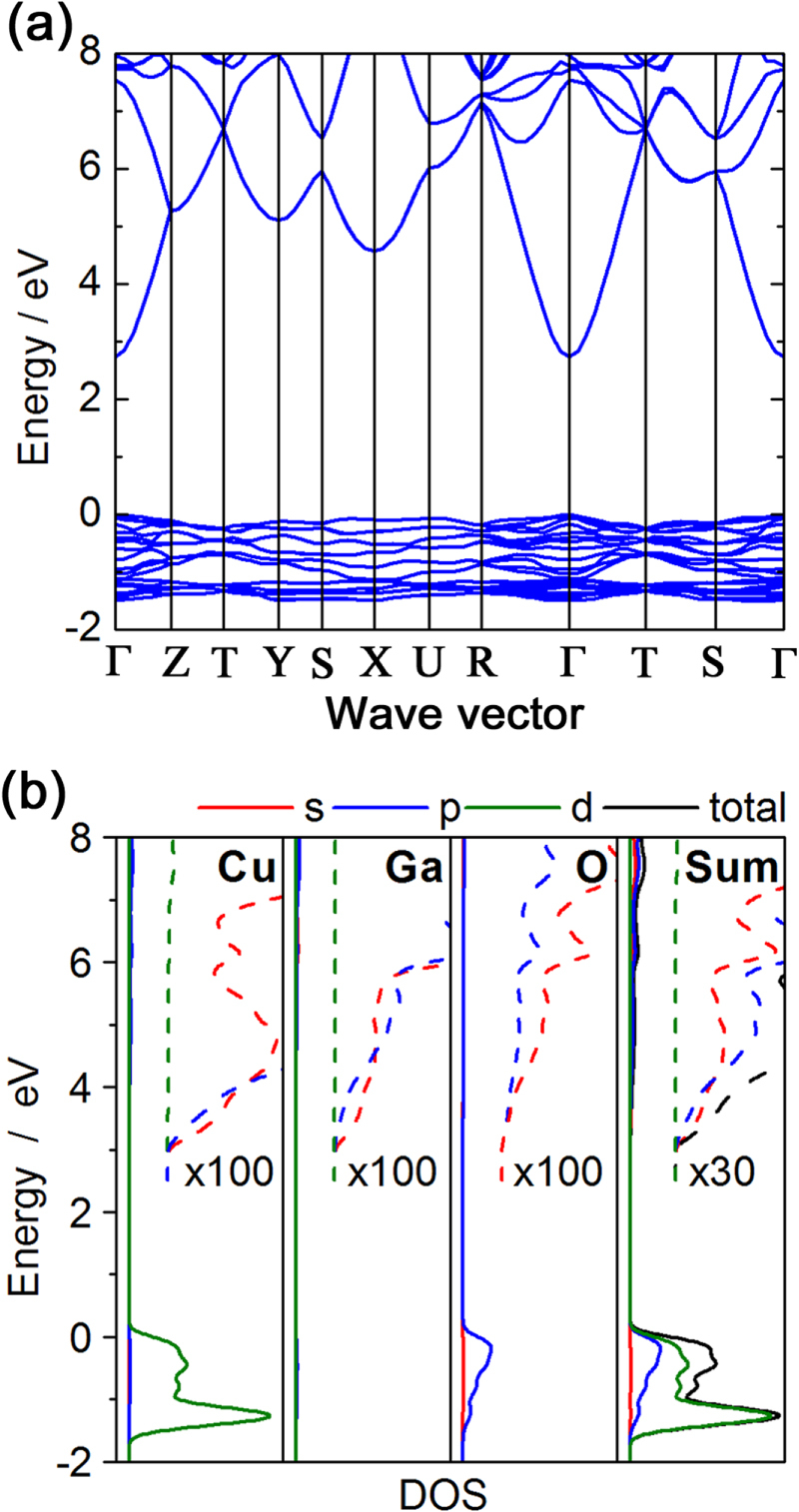
Electronic band structure of wurtzite-derived *β*-CuGaO_2_ calculated using the sX-LDA functional. (a) The band structure along the symmetry line and (b) the corresponding total and partial density of states.

Theoretical calculations of the electronic band structure, as shown in figure 7, indicate that *β*-CuGaO_2_ is a direct band gap semiconductor unlike *β*-AgGaO_2_ and *β*-AgAlO_2_ [29, 52]. The direct band gap and high density of states around the VBM because of the significant contribution of Cu 3d states enables the intense absorption of light. Because *β*-CuGaO_2_ possesses these optical features and its band gap matches the band gap required to achieve the theoretical maximum conversion efficiency for a single-junction solar cell, as shown in the inset of Figure 6 [63], it is a promising light absorbing material in thin-film solar cells, similar to CdTe and Cu(In,Ga)Se_2_.

The intentional doping of *β*-CuGaO_2_ has not been reported and, therefore, its detailed electrical transport property is not yet understood. However, the sign of the thermoelectromotive force measured at room temperature was positive. This indicates that the material is a *p*-type semiconductor without intentional doping. This is similar to copper-containing delafossite oxides [64–67]. The *p*-type electrical conduction and the small lattice mismatch of *β*-CuGaO_2_ with ZnO, i.e., 0.6% in the *ab*-plane and 1.4% along the *c*-axis are promising for the fabrication of a *p–n* heterojunction between *p*-type *β*-CuGaO_2_ and *n*-type ZnO. We are now investigating methods to control its electrical properties and to fabricate thin films of *β*-CuGaO_2_ for the development of optoelectronic devices that work in the near-infrared region.

## 5. Summary and outlook

**Figure 8.**
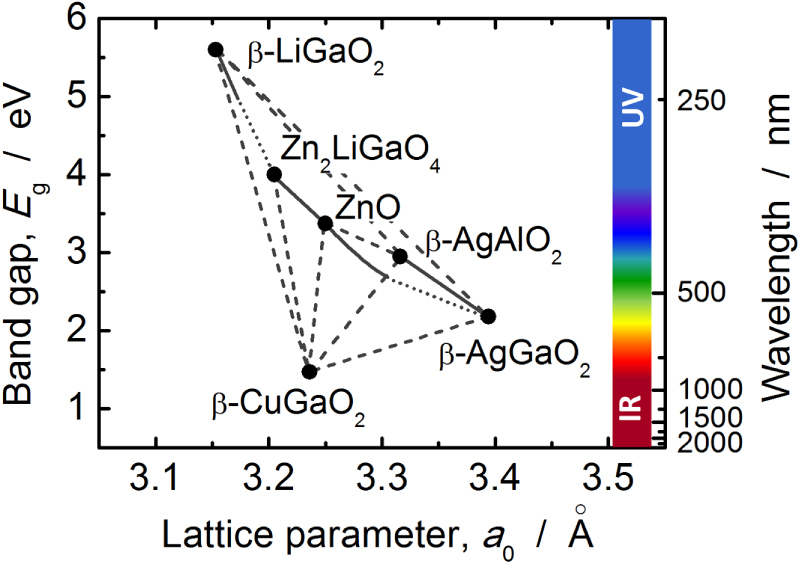
Band gap versus pseudo-wurtzite lattice parameter, *a*_0_, for binary and ternary wurtzite-type oxide semiconductors.

Wurtzite-derived ternary I–III–O_2_ semiconductors have expanded the energy range that oxide semiconductors cover as shown in figure 8. The included range is not only the UV region but also the visible and near-infrared regions. This is similar to II–VI chalcogenides and III–V pnictide semiconductors. The electronic transport properties of oxides are limited compared with chalcogenides and pnictides because of their ionic nature. However, oxides have some advantages over chalcogenides and pnictides, such as their abundance and the non-toxicity of oxygen. Additionally, oxides maintain excellent stability in air and water under ambient conditions. Consequently, oxide semiconductors are very attractive materials for optoelectronic applications that work in the visible to the near-infrared region in addition to the visible to UV region.

The *β*-NaFeO_2_ structure has lower crystal symmetry than the chalcopyrite structure. The structural deviation from the ideal wurtzite structure, i.e., the distortion of cation and oxygen tetrahedra from an ideal tetrahedron increases with an increase in the size difference between the monovalent and trivalent cations. The structural distortion was studied and is discussed in terms of crystal structural chemistry [68]. A few reports exist about the relationship between structural distortion and physical properties [62, 69]. Because the structural distortion inevitably influences the nature of the chemical bonds and eventually their physical properties, a determination of the relationship between structural features and physical properties is a valuable and important challenge.

In this paper, we focused on the band gap engineering of oxide semiconductors with wurtzite and derived structures. We thus describe oxides that contain cations of main group elements with *n*s^0^ and/or (*n* − 1)d^10^*n*s^0^ electronic configurations. In addition to these oxides, ternary oxides may contain trivalent transition metallic ions. The multiferroic ferromagnetic and ferroelectric property has been reported for *β*-NaFeO_2_ [70]. This area also needs to be developed further for wurtzite-derived I–III–O_2_ oxides.

## References

[C1] Ginley D S, Bright C (2000). MRS Bull..

[C2] Ellmer K (2012). Nat. Photon..

[C3] Minami T (2008). Thin Solid Films.

[C4] Fortunato E, Barquinha P, Martins R (2012). Adv. Mater..

[C5] Harrison W A (1980). Electronic Structure and the Properties of Solids: The Physics of the Chemical Bond.

[C6] Özgür Ü, Alivov Y I, Liu C, Teko A, Reshchikov M A, Doğan S, Vrutin V, Cho S-J, Morkoç H (2005). J. Appl. Phys..

[C7] Tsukazaki A (2005). Nat. Mater..

[C8] Sabine T M, Hogg S (1969). Acta Crystallogr. B.

[C9] Ohtomo A, Kawasaki M, Koida T, Masubuchi K, Koinuma H, Sakurai Y, Yoshida Y, Yasuda T, Segawa Y (1988). Appl. Phys. Lett..

[C10] Ryokena H, Ohashi N, Sakaguchi I, Adachi Y, Hishita S, Haneda H (2006). J. Cryst. Growth.

[C11] Kim Y-I, Seshadri R (2008). Inorg. Chem..

[C12] Azimi H, Hou Y, Brabec C J (2014). Energy Environ. Sci..

[C13] Miles R W, Zoppi G, Forbes I (2007). Mater. Today.

[C14] Bob B, Lei B, Chung C-H, Yang W, Hsu W-C, Duan H-S, Hou W W-J, Li S-H, Yang Y (2012). Adv. Energy Mater..

[C15] Riede V, Neumann H, Sharif N, Hasoon F S, Sobatta H (1989). Phys. Status. Solidi A.

[C16] Robbins M, Miksovsky M A (1972). J. Solid State Chem..

[C17] Grima-Gallardo P, Munoz M, Ruiz J, Power C, Gonzalez J, LeGodec Y, Munsch P, Itie J P, Briceno V, Briceno J M (2004). Phys. Status Solidi B.

[C18] Apple E F (1958). J. Electrochem. Soc..

[C19] Gan J N, Tauc J, Lambrecht V G, Robbins M (1975). Phys. Rev. B.

[C20] Gallardo P G (1992). Phys. Status Solidi A.

[C21] Halka V O, Olekseyuk I D, Parasyuk O V (2000). J. Alloys Compd..

[C22] Wyckoff R W G (1964). Crystal Structure.

[C23] Wyckoff R W G (1964). Crystal Structure.

[C24] Misaki T, Tsuchiya K, Sakai D, Wakahara A, Okada H, Yoshida A (2002). Phys. Status Solidi C.

[C25] Rufinus J (2011). J. Appl. Phys..

[C26] Punya A, Lambrecht W P L (2013). Phys. Rev. B.

[C27] Nicholls J F H, Gallangher H, Henderson B, Trager-Cowan C, Middleton P G, O’Donnell L P (1996). Mater. Res. Soc. Symp. Proc..

[C28] Ouyang S, Kikugawa N, Chem D, Zou Z, Ye J (2009). J. Phys. Chem. C.

[C29] Omata T, Nagatani H, Suzuki I, Kita M, Yanagi H, Ohashi N (2014). J. Am. Chem. Soc..

[C30] Chou M M C, Hang D-R, Chen C, Liao Y-H (2011). Thin Solid Films.

[C31] Ishii T, Tazoh Y, Miyazawa S (1998). J. Cryst. Growth.

[C32] Huang T, Zhou S, Teng H, Lin H, Wang J, Han P, Zhang R (2008). J. Cryst. Growth.

[C33] Ohkubo I (2002). J. Appl. Phys..

[C34] Miller R C, Nordland W A, Kolb E D, Bond W L (1970). J. Appl. Phys..

[C35] Rashkeev S N, Limpijumnong S, Lambrecht W R L (1999). J. Opt. Soc. Am. B.

[C36] Knoll P, Kuzmany H (1984). Phys. Rev. B.

[C37] Omata T, Tanaka K, Tazuke A, Nose K, Otsuka-Yao-Matsuo S (2008). J. Appl. Phys..

[C38] Omata T, Kita M, Tachibana K, Otsuka-Yao-Matsuo S (2012). J. Solid State Chem..

[C39] Omata T, Kita M, Nose K, Tachibana K, Otsuka-Yao-Matsuo S (2011). Japan. J. Appl. Phys..

[C40] Chang H-C (1966). Acta Geologica Sin..

[C41] Limpijumnong S, Lambrecht W R L, Segall B, Kim K (1997). Mater. Res. Soc. Symp. Proc..

[C42] Johnson N W, McLeod J A, Moewes A (2011). J. Phys.: Condens. Matter.

[C43] Boonchun A, Lambrecht W R L (2010). Phys. Rev. B.

[C44] Boonchun A, Lambrecht W R L (2011). Proc. SPIE.

[C45] Li Q F, Kuo J-L (2013). J. Appl. Phys..

[C46] Omata T, Tanaka K, Otsuka-Yao-Matsuo S (2011). Japan. J. Appl. Phys..

[C47] Wyckoff R W G (1964). Crystal Structure.

[C48] Vanaja K A, Ajimsha R S, Asha A S, Jayaraj M K Appl. Phys. Lett..

[C49] Maruyama Y, Irie H, Hashimoto K (2006). J. Phys. Chem. B.

[C50] Ouyang S, Li Z, Ouyang Z, Yu T, Ye J, Zou Z (2008). J. Phys. Chem. C.

[C51] Ouyang S, Zhang H, Li D, Yu T, Ye J, Zou Z (2006). J. Phys. Chem. B.

[C52] Ouyang S, Ye J (2011). J. Am. Chem. Soc..

[C53] Suzuki I, Nagatani H, Arima Y, Kita M, Omata T (2014). Thin Solid Films.

[C54] Suzuki I, Nagatani H, Arima Y, Kita M, Omata T (2013). Appl. Phys. Lett..

[C55] Anandan S, Ohashi N, Miyauchi M (2010). Appl. Catal. B.

[C56] Köhler B U, Jansen M (1986). Z. Anorg. Allg. Chem..

[C57] Shimode M, Sasaki M, Mukaida K (2000). J. Solid State Chem..

[C58] Kawazoe H, Yanagi H, Ueda K, Hosono H (2000). MRS Bull..

[C59] Yeh J J, Lindau I (1985). At. Data Nucl. Data Tables.

[C60] Vanaja K A, Ajimsha R S, Asha A S, RajeevKumar K, Jayaraj M K Thin Solid Films.

[C61] Srinivasan R, Chavillon B, Doussier-Brochard C, Cario L, Paris M, Gautron E, Deniard P, Odobel F, Jobic S (2008). J. Mater. Chem..

[C62] Nagatani H, Suzuki I, Kita M, Tanaka M, Katsuya Y, Sakata O, Miyoshi S, Yamaguchi S, Omata T (2015). Inorg. Chem..

[C63] Shockley W, Queisser H J (1961). J. Appl. Phys..

[C64] Meyer B K (2013). Semicond. Semimetals.

[C65] Kawazoe H, Yasukawa M, Hyodo H, Kurita M, Yanagi H, Hosono H (1997). Nature.

[C66] Yanagi H, Hase T, Ibuki S, Ueda K, Hosono H (2001). Appl. Phys. Lett..

[C67] Ueda K, Hase T, Yanagi H, Kawazoe H, Hosono H, Ohta H, Orita M, Hirano M (2001). J. Appl. Phys..

[C68] Li J, Sleight AW (2004). J. Solid State Chem..

[C69] Nagatani H, Suzuki I, Kita M, Tanaka M, Katsuya Y, Sakata O, Omata T (2015). J. Solid State Chem..

[C70] Viret M, Rubi D, Colson D, Lebeugle D, Forget A, Bonville P, Dhalenne G, Saint-Martin R, Andre G, Ott F (2012). Mater. Res. Bull..

